# Comparison of Integrase Strand Transfer Inhibitors (INSTIs) and Protease-Boosted Inhibitors (PIs) on the Reduction in Chronic Immune Activation in a Virally Suppressed, Mainly Male Population Living with HIV (PLWH)

**DOI:** 10.3390/medicina60020331

**Published:** 2024-02-15

**Authors:** Thomas Nitsotolis, Konstantinos G. Kyriakoulis, Anastasios Kollias, Alexia Papalexandrou, Helen Kalampoka, Elpida Mastrogianni, Dimitrios Basoulis, Mina Psichogiou

**Affiliations:** 1 3rd Department of Internal Medicine, School of Medicine, Sotiria Hospital, National and Kapodistrian University of Athens, 11527 Athens, Greece; tomnitsotolis@gmail.com (T.N.); konkyriakoulis@gmail.com (K.G.K.); taskollias@gmail.com (A.K.); 2 Nephrological Center “Frontis” of Piraeus, 18757 Piraeus, Greece; alexiapapalexandrou@gmail.com; 3 Department of Clinical Biochemistry, University General Hospital “ATTIKO”, National and Kapodistrian University of Athens Medical School, 12462 Athens, Greece; elenikalamp@yahoo.gr; 4 1st Department of Internal Medicine, Laiko General Hospital, National and Kapodistrian University of Athens Medical School, 11527 Athens, Greece; elpidamastrogianni@gmail.com (E.M.); dimitris.bassoulis@gmail.com (D.B.)

**Keywords:** chronic immune activation, IL-6, intestinal bacterial translocation, I-FABP, INSTIs, PIs/boosted, LBP, sCD14, SNAEs, SuPAR

## Abstract

*Background and Objectives:* The success of combined antiretroviral therapy (cART) has led to a dramatic improvement in the life expectancy of people living with HIV (PLWH). However, there has been an observed increase in cardiometabolic, bone, renal, hepatic, and neurocognitive manifestations, as well as neoplasms, known as serious non-AIDS events/SNAEs, compared to the general population of corresponding age. This increase is linked to a harmful phenomenon called inflammaging/immunosenescence, which is driven by chronic immune activation and intestinal bacterial translocation. In this study, we examined immunological and metabolic parameters in individuals receiving current cART. *Materials and Methods:* The study was conducted at Laiko General Hospital in Athens, Greece. Plasma concentrations of sCD14, IL-6, SuPAR, I-FABP, and LBP were measured in virally suppressed PLWH under cART with at least 350 CD4 lymphocytes/μL. We compared these levels between PLWH receiving integrase strand transfer inhibitors (INSTIs) and protease inhibitors (PIs) and attempted to correlate them with chronic immune activation and metabolic parameters. *Results:* Data from 28 PLWH were analyzed, with a mean age of 52 and 93% being males. Among the two comparison groups, IL-6 levels were higher in the PIs group (5.65 vs. 7.11 pg/mL, *p* = 0.03). No statistically significant differences were found in the other measured parameters. A greater proportion of PLWH under INSTIs had normal-range LBP (33% vs. 0%, *p* = 0.04). When using inverse probability of treatment weighting, no statistically significant differences in the measured parameters were found between the two groups (sCD14 *p* = 0.511, IL-6 *p* = 0.383, SuPAR *p* = 0.793, I-FABP *p* = 0.868, and LBP *p* = 0.663). Glucose levels were found to increase after viral suppression in the entire sample (92 mg/dL vs. 98 mg/dL, *p* = 0.009). Total (191 mg/dL vs. 222 mg/dL, *p* = 0.005) and LDL cholesterol (104 mg/dL vs. 140 mg/dL, *p* = 0.002) levels were higher in the PIs group. No significant differences were observed in liver and renal function tests. *Conclusions:* Further investigation is warranted for PLWH on cART-containing INSTI regimens to explore potential reductions in chronic immune activation and intestinal bacterial translocation.

## 1. Introduction

The administration of highly effective combined antiretroviral therapy (cART) has led to viral suppression at levels undetectable by even the most modern and sensitive molecular methods [1,2]. Despite receiving cART, some individuals still experience incomplete mitigation of immune activation and intestinal bacterial translocation markers [1,2,3]. This suggests that the therapy’s effectiveness in reducing these markers may vary among patients, highlighting the need for further research and potentially alternative treatment approaches [1,2,3].

Despite effective cART administration, levels of IL-6, monocyte cell activation markers (soluble receptor molecule/sCD14 and soluble urokinase plasminogen activator receptor/SuPAR), and biomarkers for intestinal bacterial translocation (intestinal fatty acid binding protein/I-FABP and lipopolysaccharide binding protein/LBP) remain elevated compared to the general population [3,4,5].

Individuals under cART experience serious non-AIDS events (SNAEs), such as non-AIDS malignancies, cardiovascular events, renal and hepatic diseases, bone disorders, and neurocognitive impairment [6,7,8]. SNAEs are considered the main causes of morbidity and mortality among PLWH in the cART era [6,7,8].

Chronic aberrant inflammation and intestinal bacterial translocation contribute to atherosclerosis, tumor development, and liver fibrosis [7,8,9,10]. Elevated levels of biomarkers associated with chronic inflammation and bacterial translocation increase the risk of cardiovascular diseases and malignancies, sharing similar pathophysiological mechanisms with the general population but not identical ones [3,4,9,11].

Factors contributing to the emergence of SNAEs include HIV’s direct effect and related immunodeficiency before cART, underlying comorbidities, coinfections, and drug toxicities [12].

In this article, we aim to compare the potential immunological pleiotropic effects and metabolic parameters in PLWH receiving different cART regimens.

## 2. Materials and Methods

### 2.1. Study Design and Setting

A pilot study was conducted in the Infectious Disease Department of the First University Department of Internal Medicine at Laiko General Hospital in Athens, Greece, in January 2022. Ethical approval was obtained from the Laiko Hospital Scientific Committee (ID 13494, 24 October 2017), and all participants provided informed consent.

### 2.2. Participants

Twenty-eight consecutive HIV-positive individuals received cART based on either INSTIs regimens or PI/boosted regimens in combination with two nucleoside/nucleotide reverse transcriptase inhibitor/N(t)RTI regimens as the backbone therapy.

Patients were eligible for inclusion if they were (i) adults >18 years old, (ii) diagnosed with HIV infection, (iii) under cART, and (iv) at least six months after complete viral suppression with an absolute peripheral blood count of CD4+ lymphocytes > 350/μL.

Exclusion criteria were (i) incomplete adherence to cART, (ii) known history of solid organ or hematopoietic malignancies, (iii) autoimmune, autoinflammatory, or other rheumatic disorders, (iv) HCV/HBV/HDV coinfections or liver cirrhosis/severe hepatic dysfunction/failure, (v) chronic kidney disease (estimated glomerular filtration rate/eGFR < 60 mL/min), (vi) alcohol abuse or use of other toxic substances, (vii) acute or chronic respiratory failure (type 1 or type 2), (viii) heart failure according to NYHA stages III–IV, (ix) inflammatory bowel disease or celiac disease, (x) history of gastrointestinal surgery, and (xi) use of immunosuppressive or nonsteroidal anti-inflammatory drugs.

### 2.3. Study Time Points and Data Collection

Individuals were assessed for eligibility and recruited during the recruitment visit, where blood was sampled, and data were collected from their medical files regarding three past time points: (i) HIV diagnosis, (ii) cART initiation, and (iii) 1 year after cART initiation.

**Figure 1:**
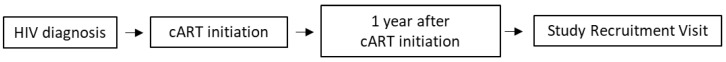
Brief visual overview of study timeline, time points, and procedures.

A brief visual overview of the study timeline, visits, and procedures is shown in Figure 1. A detailed description of variables collected during the study time points is shown in Table 1 and Table 2.

### 2.4. Outcomes

The primary outcome of our study was the comparison of specific biomarkers such as sCD14, IL-6, SuPAR, I-FABP, and LBP between individuals receiving INSTI regimens vs. those receiving PI/boosted regimens during recruitment visits. Normal values of the above laboratory parameters were based on published literature [13,14,15,16].

Secondary analyses included comparisons of the rest of laboratory values during the recruitment visit (undetectable viral load and CD4+ > 350/μL) and the comparison of past laboratory parameters at the time of HIV diagnosis, cART initiation, and after 1 year of initiation of cART between the two study treatment subgroups (INSTI vs. PI/boosted regimens).

### 2.5. Methods for Determining the Measured Plasma Biomarkers

Patients were subjected to blood sampling after overnight fasting. Upon venipuncture, 1 mL of blood was centrifuged at 3000× *g*, 5 min at 22 °C, and plasma was collected and kept at −80 °C until the determination of cytokines and other biomarkers plasma levels. 

The plasma levels of IL-6, sCD14, LBP, SuPAR, and I-FABP were determined using commercially available kits (details in Supplementary File, Table S1).

### 2.6. Statistical Analysis

The distributions of continuous variables were a priori considered non-normal due to the limited sample size. Medians and ranges were used to describe continuous variables. Absolute values and percentages were used to describe categorical variables. Comparisons between subgroups were performed using the Mann–Whitney U or Fisher’s exact test, as appropriate. The Wilcoxon signed-rank test was used for comparisons of longitudinal data (e.g., comparisons of laboratory values between different time points).

Due to inherent biases in treatment choices (e.g., indication bias) and differences in baseline variables (e.g., AIDS or infection at diagnosis), the effect of the treatment group was also investigated in a secondary alternative analysis after performing inverse probability treatment weighting (IPTW) to assess the average treatment effect of INSTI regimens vs. PI/boosted regimens. IPTW assigns weights to each case based on a propensity score. This approach avoids individual exclusion as in propensity score matching but creates a synthetic sample with an independent distribution of baseline covariates and treatment assignment [17]. The propensity score was calculated using the following variables: age, gender, CD4+ lymphocyte count, HIV RNA viral load, and presence of AIDS at diagnosis. The analysis was performed using the Stata v16 (StataCorp LLC, College Station, TX, USA) statistical package. Statistical significance was set at a two-sided 0.05 level.

## 3. Results

### 3.1. Baseline Characteristics of Included Participants (PLWH), which Were Collected from Their Medical Records (during the Recruitment Visit)

Twenty-eight participants were included in the present cohort. Fifteen individuals were receiving INSTI-based cART regimens, while the remaining individuals were on PI/boosted-based regimens. The baseline characteristics of the included patients are shown in detail in Table 3. The two treatment groups were equally balanced in terms of sex and age. The median time interval from HIV diagnosis to cART initiation was 2 months (5 days–11 years) with no difference between treatment groups. The median time interval from HIV diagnosis to recruitment was 7 years (range: 1–21), with no difference observed between treatment groups. Patients receiving INSTI regimens had a higher BMI compared with patients receiving PI/boosted regimens (median 29 kg/m^2^ vs. 22 kg/m^2^) and were more likely to be current smokers (47% vs. 0%). Conversely, patients receiving PI-boosted regimens presented with more severe disease at diagnosis (C3 stage 61% vs. 7%) (Table 3).

### 3.2. Primary Analysis/Outcome of Interest

Between the two treatment groups, PLWH receiving INSTI-based regimens had lower IL-6 titers compared with PI/boosted regimens (5.65 vs. 7.11 pg/mL, *p* = 0.03) (Table 4). There were no statistically significant differences among the other measured parameters between the two groups. However, a greater proportion of PLWH who were under INSTIs had a normal range of LBP compared with the other group (33% vs. 0%, *p* = 0.04). as shown in Table 5, where an analysis of normal/abnormal status based on cut-off values is presented.

Using IPTW, there were no statistically significant differences in measured parameters between the two groups (sCD14 *p* = 0.511, IL-6 *p* = 0.383, SuPAR *p* = 0.793, I-FABP *p* = 0.868, and LBP *p* = 0.663).

### 3.3. Secondary Analyses

#### 3.3.1. Comparison of Resting Immune Parameters during the Recruitment Visit

**Figure 2:**
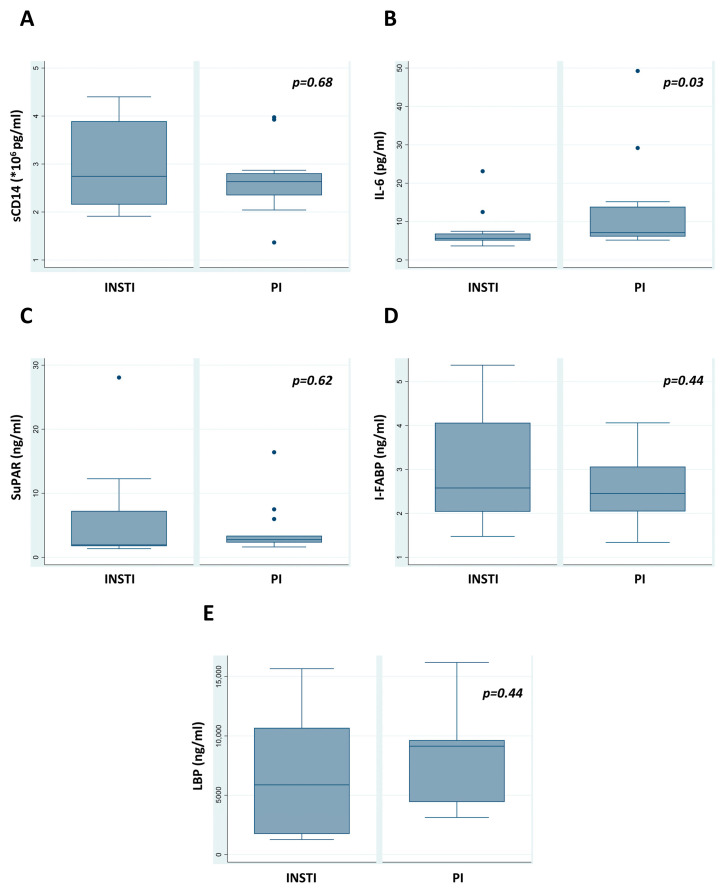
Comparison of concentrations (**A**) sCD14, (**B**) IL-6, (**C**) SuPAR, (**D**) I-FABP, and (**E**) LBP postviral suppression. The *p*-value was significant for IL-6 comparison (*p* = 0.03). Outlier values are shown with dots.

The number of CD4+ cells was significantly higher in patients receiving INSTI regimens (726 vs. 495 cells/μL, *p* = 0.01); however, no difference was detected when CD4+ cell differences (values at recruitment visit minus at HIV diagnosis) were considered (CD4+ cell difference 402 vs. 390 for INSTI vs. PI/boosted regimens, respectively, *p* = 0.93). The CD4+/CD8+ ratio did not present a significant difference (Table 4). Respective box plots are shown in Figure 2.

#### 3.3.2. Comparison of Metabolic Parameters during the Recruitment Visit

Total and LDL cholesterol levels were higher in patients receiving PI/boosted regimens (total cholesterol: 191 mg/dL vs. 222 mg/dL, *p* = 0.005 and LDL cholesterol: 104 mg/dL vs. 140 mg/dL, *p* = 0.002) (Table 4). No significant differences were apparent in other laboratory parameters, such as those used to examine liver or kidney function (Table 4).

#### 3.3.3. Comparison of Longitudinal Laboratory Immune Parameters

Regarding the HIV diagnosis versus the recruitment visit comparison, all viral parameters improved over time in both treatment groups (Table 6). CD4+ T cell numbers ranged from 351 to 2143 cells/μL. CD8+ CTL numbers were increased only in the case of patients receiving PI-boosted regimens; however, the CD4+/CD8+ ratio was uniformly improved in both groups, with a greater tendency to normalize in subjects receiving INSTI regimens.

#### 3.3.4. Comparison of Longitudinal Laboratory Metabolic Parameters

Uric acid levels were significantly higher in the INSTIs treatment group, while a slightly greater but nonsignificant increase was observed in the PIs/boosted group (HIV diagnosis vs. recruitment visit) (Table 6).

**Figure 3:**
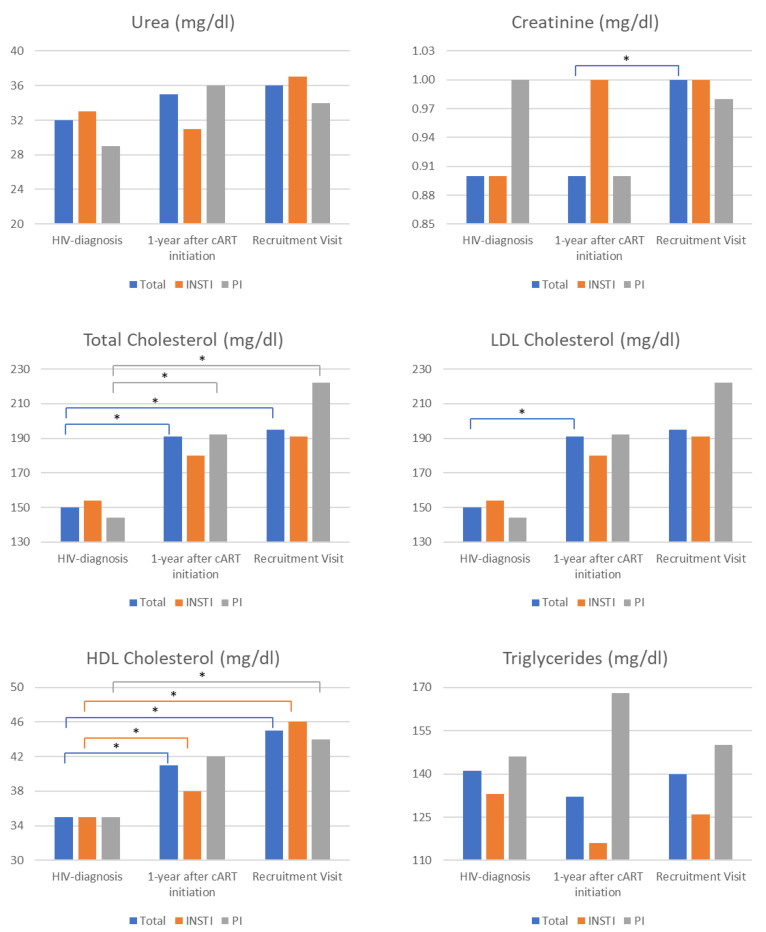
Comparison of metabolic laboratory findings was conducted in three different instances: (1) during HIV diagnosis, (2) 1 year after cART (combination antiretroviral therapy) initiation, (3) during the recruitment visit. The metabolic parameters measured in all three instances included urea, creatinine, and cholesterol. The asterisk denotes a statistically significant comparison after applying the Bonferroni correction for multiple comparisons. The color of the respective line below the asterisk indicates the comparison to which the statistical significance (asterisk) refers. E.g., in the case of creatinine, significance (asterisk) was observed only for the comparison of 1 year after cART initiation vs. recruitment visit levels (blue line).

Comparisons of metabolic parameters available during three time points (HIV diagnosis, 1 year after cART initiation, and during the recruitment visit) indicated a significant increase in total creatinine from diagnosis and 1 year after cART initiation to the recruitment visit (Figure 3). A similar trend was observed for the INSTIs group; however, statistical significance was not achieved, probably due to the limited sample size. Total cholesterol levels were generally increased but more consistently in patients receiving PI-boosted regimens. LDL cholesterol levels presented a similar increasing trend, however, reaching statistical significance only for total LDL cholesterol levels between HIV diagnosis and 1 year after cART initiation evaluations. HDL cholesterol levels were uniformly increased in all patients. Triglycerides were unaffected during follow-up visits (Figure 3).

## 4. Discussion

The main findings of the present retrospective pilot cohort study were (i) a difference in IL-6 levels (5.65 vs. 7.11 pg/mL, *p* = 0.03 for INSTI vs. PI regimens, respectively), (ii) trends toward reduced levels of LBP, and (iii) normalization of the CD4+/CD8+ ratio in INSTI regimens recipients.

The production of IL-6 during inflammatory conditions and infections is induced mainly through the stimulation of innate immune cells by IL-1 or tumor necrosis factor-a (TNF-a) or through the stimulation of TLR receptors (Toll-like receptors) after binding of PAMPs (pathogen-associated molecular patterns) or DAMPs (danger-associated molecular patterns/alarmins) [18,19].

IL-6 is a 25 kDa glycopeptide consisting of 184 amino acids [18]. It is produced by monocytes, macrophages, T cells, B cells, hepatocytes, endothelial cells, fibroblasts, keratinocytes, mesangial kidney cells, adipocytes, and certain tumor cells [18].

In PLWH, even if complete viral suppression has been established with cART administration, IL-6 levels are never normalized to the previously basal levels. IL-6 serves as a useful biomarker for chronic and aberrant immune activation and the likelihood of SNAEs, specifically severe cardiovascular events [18].

Despite the small number of participants, in which the majority consisted of males, a particularly interesting finding of our study was that PLWH on cART-containing INSTI regimens exhibited a slight trend toward lower concentrations of LBP in peripheral plasma. This finding certainly deserves further investigation for any potential reduction in intestinal bacterial translocation. Whether these findings would be the same in a predominantly female population remains uncertain and requires further investigation.

SuPAR is the soluble form of urokinase plasminogen activator receptor (uPAR), a protein that attaches to cell membranes using glycosyl phosphatidyl (GPI) moieties [20]. It is present in immune cells, fibroblasts, and myocardial cells [20]. uPAR plays various roles in cell proliferation, angiogenesis, and fibrinolysis [20]. In inflammatory conditions, the GPI moiety breaks down, causing the release of soluble SuPAR [20].

In our study, none of the above was proven, regardless of the combined cART obtained, and a possible explanation is the small number of participants.

Intestinal bacterial translocation is a critical guide for the activation of the immune system (innate and adaptive with aberrant pathways), and biomarkers that at least indirectly express it, as identified in the systemic circulation, are often associated with imminent disease progression [1]. Since the disruption of the intestinal epithelial barrier leads to microbial translocation, the assessment of enterocyte damage is a reliable indicator of its approach [1]. A biomarker of enterocyte injury and loss is the elevated plasma levels of intestinal fatty acid binding protein (I-FABP), which is expressed exclusively by enterocytes [1]. I-FABP is a cytoplasmic protein that is specifically expressed in the mature enterocytes of the small intestine in the jejunum [21]. 

Higher I-FABP levels in PLWH indicate intestinal epithelial injury [21]. The dysfunctional gastrointestinal mucosa in PLWH is attributed to the early loss of Th17 mucosal cells and impaired epithelial integrity, resulting in increased microbial translocation [21]. Elevated levels of fatty acid binding intestinal protein (I-FABP) in the systemic circulation are associated with higher morbidity and mortality, as well as lower absolute counts of CD4+ T cells [21].

Our findings support the existing literature on I-FABP levels and indicate that intestinal epithelial barrier damage remains unresolved despite effective cART. It is important to note that we did not measure any other biomarkers of intestinal epithelial barrier injury. This omission prevents us from determining whether INSTI or PI-boosted regimens are more effective in mitigating this harmful pathophysiological phenomenon.

Elevated levels of lipopolysaccharide binding protein (LPB) are due to intestinal microbial translocation [22]. Such changes have often been documented in PLWH and serve as critical serum biomarkers of virus-associated chronic and aberrant immune activation [22]. As described above, the presence of microbial products in the systemic circulation leads to the activation of immune cells (mainly of innate immunity) through TLR–TLR ligand interactions [22,23].

LBP is unique in that it binds to the LPS micelle and delivers a monomer to the CD14 coreceptor, which enhances the TLR4/MD-2 response [22,23]. Thus, it could be characterized as a soluble pattern recognition receptor (PRR) [23]. Due to its stable plasma levels, unlike LPS, it could be a reliable biomarker of intestinal bacterial translocation [24].

Another interesting finding of the present study that deserves further investigation is that PLWH on cART-containing INSTI regimens also showed a better trend in the normalization of the CD4+/CD8+ ratio, getting closer to the value of 1. This ratio may represent the combined effects of inflammation and immunological changes called inflammaging [25]. Although the mechanisms underlying partial correction of the CD4+/CD8+ ratio and persistently elevated CD8+ T cell count in long-term treated patients remain poorly understood, it has been recently indicated that patients with optimal CD4+ T cell recovery and low CD4+/CD8+ ratio still harbor increased immune activation, an immune senescent phenotype, and have a higher risk of SNAEs and mortality [25].

Until ‘functional cures’ aiming for durable viral control in the absence of the administration of cART and ‘sterilizing cures’ aiming for the more difficult-to-realize objective of complete viral eradication can be applied at a practical clinical level, we need to explore and exploit the advantages of existing cART, especially perhaps those that include INSTI regimens [19].

One of the main drawbacks of our study was that it was conducted during the COVID-19 pandemic, which had obvious effects on the recruitment of a sufficient number of participants due to the inability to have free and unhindered access to healthcare facilities. This significantly reduced the number of subjects enrolled in the study and hindered the acquisition of the extremely basic biomarkers that reflect chronic immune activation and intestinal bacterial translocation.

Moreover, due to the single-center nature of our study in a solely Greek population, making generalizations about our findings is dubious. Selection bias when choosing cART regimens might also influence our findings, which we attempted to address with the IPTW analysis.

## 5. Conclusions

Despite the small number of PLWH participating in the present study, the fact that most of them were males, as well as the limited number of measured biomarkers, there is a trend toward reduced levels of IL-6 and normalization of LBP, as well as the CD4+/CD8+ ratio, in cART recipients receiving INSTI-based regimens.

Based on the above larger and extended sample size, a greater number of participants and measured biomarkers are required to substantiate the potential pleiotropic effect of INSTI regimens in reducing chronic aberrant immune activation and intestinal bacterial translocation.

## Figures and Tables

**Table 1:** Overview of study visits and procedures (patient details/history).

Assessment	HIV Diagnosis	Recruitment Visit
Selection Criteria		✓
Medical History		✓
Clinical Examination		✓

**Table 2:** Overview of study visits and procedures (laboratory tests evaluation).

	HIV Diagnosis	Recruitment Visit
CD4+	✓	✓
CD8+	✓	✓
CD4+/CD8+	✓	✓
NK cells	✓	✓
HIV RNA copies/ml	✓	✓
Glucose	✓	✓
Urea/creatinine	✓	✓
Cholesterol	✓	✓
Liver function	✓	✓
Uric acid	✓	✓
sCD14		✓
IL-6		✓
SuPAR		✓
I-FABP		✓
LBP		✓

cART, combination antiretroviral therapy; I-FABP, intestinal fatty acid binding protein; IL-6, interleukin-6; LBP, lipopolysaccharide binding protein; SuPAR, soluble urokinase plasminogen activator receptor. Data on glucose, urea, creatinine, and cholesterol were also collected 1 year after cART initiation.

**Table 3:** Baseline characteristics of individuals that were collected from their medical records (during the recruitment visit).

Assessment	Overall	INSTIs	PIs	*p*-Value
***n***	28	15	13	-
**Males**	26 (93)	15 (100)	11 (85)	0.21
**Age**	52 (32–70)	52 (32–67)	53 (33–70)	0.47
**BMI**	25 (13–39)	29 (25–39)	22 (13–29)	**0.01**
**Ethnicity**				0.27
European	27 (96)	15 (100)	12 (92)	
African	1 (4)	0 (0)	1 (8)	
**Greek nationality**	25 (89)	15 (100)	10 (77)	0.09
**MSM**	19 (70)	11 (79)	8 (62)	0.42
**Sexual practices**				0.64
Heterosexual	8 (30)	3 (21)	5 (38)	
Homosexual	16 (59)	9 (64)	7 (54)	
Bisexual	3 (11)	2 (14)	1 (8)	
**Years from HIV diagnosis to recruitment visit**	7 (1–21)	7 (1–15)	7 (2–21)	0.93
**Disease stage at diagnosis**				**0.003**
A1	4 (14)	4 (27)	0 (0)	
A2	8 (29)	7 (46)	1 (8)	
B1	2 (7)	1 (7)	1 (8)	
B3	5 (18)	2 (13)	3 (23)	
C3	9 (32)	1 (7)	8 (61)	
**AIDS or infection at diagnosis**	9 (32)	1 (7)	8 (62)	**0.004**
**STIs at diagnosis**	8 (29)	5 (33)	3 (23)	0.69
**Smoking at diagnosis**				**0.006**
Never	15 (54)	7 (47)	8 (62)	
Past	6 (21)	1 (6)	5 (38)	
Current	7 (25)	7 (47)	(0)	
**Alcohol at diagnosis**				0.06
Never	17 (61)	7 (47)	10 (77)	
Little	8 (28)	7 (47)	1 (8)	
Moderate	3 (11)	1 (6)	2 (15)	
**Hypertension stage**				1.00
High normal	3 (11)	2 (13)	1 (8)	
Stage 1	2 (7)	1 (7)	1 (8)	
Stage 2	1 (4)	1 (7)	0 (0)	
**Diabetes**				0.40
Prediabetes	7 (25)	5 (33)	2 (15)	
Diabetes	1 (4)	0 (0)	1 (8)	
**Coronary artery disease**	1 (4)	1 (7)	0 (0)	1.00
**Laboratory tests at diagnosis**				
CD4+ (cells/µL)	222 (1–877)	397 (47–877)	64 (1–629)	**<0.001**
CD8+ (cells/µL)	881 (50–8770)	1190 (563–8770)	365 (50–1648)	**0.002**
CD4/CD8 ratio	0.19 (0.01–1.25)	0.25 (0.04–1.25)	0.11 (0.01–1.00)	**0.04**
NK (cells/µL)	131 (6–623)	134 (38–623)	102 (6–286)	0.37
HIV RNA (copies/mL)	97,763 (240–11 × 10^6^)	65,800 (3500–11 × 10^6^)	211,100 (240–5.4 × 10^6^)	0.36
Urea (mg/dL)	32 (14–56)	33 (26–56)	29 (14–51)	0.18
Creatinine (mg/dL)	0.9 (0.6–1.2)	0.9 (0.7–1.2)	1.0 (0.6–1.2)	0.69
Total cholesterol (mg/dL)	150 (79–255)	154 (100–255)	144 (79–212)	0.27
LDL cholesterol (mg/dL)	107 (31–178)	96 (40–178)	111 (31–148)	0.93
HDL cholesterol (mg/dL)	35 (15–57)	35 (15–47)	35 (27–57)	0.91
Triglycerides (mg/dL)	141 (46–282)	133 (46–282)	146 (87–177)	0.71
ALT (IU/L)	24 (7–359)	24 (10–359)	21 (7–108)	0.38
AST (IU/L)	23 (15–295)	25 (15–295)	20 (15–57)	0.34
gGT (IU/L)	27 (4–112)	27 (4–112)	26 (13–73)	0.67
ALP (IU/L)	77 (45–325)	77 (47–325)	77 (45–153)	0.62
Total bilirubin (mg/dL)	0.38 (0.17–1.36)	0.38 (0.17–1.36)	0.40 (0.27–1.10)	0.88
Uric acid (mg/dL)	4.8 (3.6–7.5)	5.5 (3.9–7.3)	4.7 (3.6–7.5)	0.86

AIDS, acquired immune deficiency syndrome; ALT, alanine transaminase; ALP, alkaline phosphatase; AST, aspartate aminotransferase; HDL, high-density lipoprotein; gGT, gamma-glutamyl transpeptidase; INSTIs, integrase strand transfer inhibitors; LDL, low-density lipoprotein; MSM, men who have sex with men; NK, natural killer cells; PIs/boosted, protease inhibitors/boosted; STIs, sexually transmitted infections. The *p*-value for comparison between individuals receiving INSTI vs. PI/boosted regimens; significant *p*-values are in bold.

**Table 4:** Laboratory findings of individuals during the recruitment visit (postviral suppression).

Assessment	Overall *n* = 28	INSTIs *n* = 15	PIs *n* = 13	*p*-Value
CD4+ (cells/µL)	665 (351–2143)	726 (383–2143)	495 (351–988)	**0.01**
CD8+ (cells/µL)	895 (292–1984)	882 (445–1984)	908 (292–1870)	0.96
CD4+/CD8+ ratio	0.73 (0.24–2.43)	0.99 (0.28–2.43)	0.53 (0.24–2.22)	0.07
NK (cells/µL)	288 (92–844)	265 (92–844)	336 (113–569)	0.79
HIV RNA (copies/mL)	20 (20–40)	20 (20–37)	20 (20–40)	0.22
Urea (mg/dL)	36 (17–66)	37 (26–56)	34 (17–66)	0.26
Creatinine (mg/dL)	1.0 (0.6–1.9)	1.0 (0.8–1.3)	0.98 (0.6–1.9)	0.66
Total cholesterol (mg/dL)	195 (127–302)	191 (127–246)	222 (156–302)	**0.005**
LDL cholesterol (mg/dL)	121 (73–186)	104 (73–153)	140 (93–186)	**0.002**
HDL cholesterol (mg/dL)	45 (29–74)	46 (30–74)	44 (29–65)	0.60
Triglycerides (mg/dL)	140 (49–398)	126 (49–275)	150 (85–398)	0.13
Glucose (mg/dL)	98 (66–175)	106 (66–133)	96 (73–175)	0.47
ALT (IU/L)	21 (10–90)	28 (10–90)	17 (12–88)	0.22
AST (IU/L)	22 (13–63)	27 (15–63)	20 (13–33)	0.09
gGT (IU/L)	24 (6–178)	29 (6–178)	23 (17–87)	1.00
ALP (IU/L)	76 (37–131)	69 (37–131)	82 (58–131)	0.21
Total bilirubin (mg/dL)	0.50 (0.21–1.28)	0.70 (0.21–1.28)	0.47 (0.24–1.23)	0.39
Uric acid (mg/dL)	6.0 (3.8–9.3)	5.6 (3.8–9.3)	6.1 (3.8–9.1)	0.95
sCD14 (×10^6^ pg/mL)	2.64 (1.37–4.40)	2.74 (1.91–4.40)	2.63 (1.37–3.97)	0.68
IL-6 (pg/mL)	6.29 (3.66–49.24)	5.65 (3.66–23.12)	7.11 (5.18–49.24)	**0.03**
SuPAR (ng/mL)	2.52 (1.34–28.07)	1.93 (1.34–28.07)	2.74 (1.61–16.40)	0.62
I-FABP (ng/mL)	2.52 (1.34–5.38)	2.58 (1.48–5.38)	2.45 (1.34–4.06)	0.44
LBP (ng/mL)	7677.9 (1265.5–16,193)	5875.1 (1265.5–15,667.5)	9137.7 (3129.4–16,193)	0.44

ALT, alanine transaminase; ALP, alkaline phosphatase; AST, aspartate aminotransferase; HDL, high-density lipoprotein; gGT, gamma-glutamyl transpeptidase; I-FABP, intestinal fatty acid binding protein; IL-6, interleukin 6; INSTIs, integrase strand transfer inhibitors; LDL, low-density lipoprotein; NK, natural killer cells; PIs/boosted, protease inhibitors/boosted; LBP, lipopolysaccharide-binding protein; SuPAR, soluble urokinase plasminogen activator receptor. The *p*-value for the comparison between INSTI versus PI/boosted treated individuals; significant *p*-values are in bold.

**Table 5:** Comparison of the percentage of normal status regarding sCD14, IL-6, SuPAR, I-FABP, and LBP values during the recruitment visit.

Assessment	Overall *n* = 28	INSTIs *n* = 15	PIs *n* = 13	*p*-Value
sCD14 (×10^6^ pg/mL) (<1.1)	0 (0)	0 (0)	0 (0)	NA
IL-6 (pg/mL) (<0.8)	0 (0)	0 (0)	0 (0)	NA
SuPAR (ng/mL) (<3)	18 (64)	9 (60)	9 (69)	0.71
I-FABP (ng/mL) (<2.1)	9 (32)	4 (27)	5 (38)	0.69
LBP (ng/mL) (<1700)	5 (18)	5 (33)	0 (0)	**0.04**

I-FABP, intestinal fatty acid binding protein; IL-6, interleukin 6; INSTIs, integrase strand transfer inhibitors; NA, not applicable; PIs/boosted, protease inhibitors/boosted; LBP, lipopolysaccharide binding protein; SuPAR, soluble urokinase plasminogen activator receptor. Cut-off values used for the definition of normal status are reported in parentheses in the first column after the measurement units. The *p*-value for the comparison between INSTI versus PI/boosted treated individuals; significant *p*-values are in bold.

**Table 6:** Comparison of laboratory findings available at HIV diagnosis vs. during the recruitment visit.

Assessment	Overall *n* = 28			INSTIs *n* = 15			PIs *n* = 13		
	HIV Diagnosis	Recruitment Visit	*p*	HIV Diagnosis	Recruitment Visit	*p*	HIV Diagnosis	Recruitment Visit	*p*-Value
CD4+ (cells/µL)	222 (1–877)	665 (351–2143)	**<0.001**	397 (47–877)	726 (383–2143)	**<0.001**	64 (1–629)	495 (351–988)	**<0.001**
CD8+ (cells/µL)	881 (50–8770)	895 (292–1984)	0.88	1190 (563–8770)	882 (445–1984)	**0.01**	365 (50–1648)	908 (292–1870)	0.07
CD4+/CD8+ ratio	0.19 (0.01–1.25)	0.73 (0.24–2.43)	**<0.001**	0.25 (0.04–1.25)	0.99 (0.28–2.43)	**<0.001**	0.11 (0.01–1.00)	0.53 (0.24–2.22)	**<0.001**
NK (cells/µL)	131 (6–623)	288 (92–844)	**<0.001**	134 (38–623)	265 (92–844)	**0.002**	102 (6–286)	336 (113–569)	**0.003**
HIV RNA (copies/mL)	97,763 (240–11 × 10^6^)	20 (20–40)	**<0.001**	65,800 (3500–11 × 10^6^)	20 (20–37)	**<0.001**	211,100 (240–5.4 × 10^6^)	20 (20–40)	**<0.001**
ALT (IU/L)	24 (7–359)	21 (10–90)	0.09	24 (10–359)	28 (10–90)	0.31	21 (7–108)	17 (12–88)	0.15
AST (IU/L)	23 (15–295)	22 (13–63)	0.36	25 (15–295)	27 (15–63)	0.69	20 (15–57)	20 (13–33)	0.26
gGT (IU/L)	27 (4–112)	24 (6–178)	0.65	27 (4–112)	29 (6–178)	0.94	26 (13–73)	23 (17–87)	0.51
ALP (IU/L)	77 (45–325)	76 (37–131)	0.43	77 (47–325)	69 (37–131)	0.11	77 (45–153)	82 (58–131)	0.37
Total bilirubin (mg/dL)	0.38 (0.17–1.36)	0.50 (0.21–1.28)	0.22	0.38 (0.17–1.36)	0.70 (0.21–1.28)	0.41	0.40 (0.27–1.10)	0.47 (0.24–1.23)	0.34
Uric acid (mg/dL)	4.8 (3.6–7.5)	6.0 (3.8–9.3)	**<0.001**	5.5 (3.9–7.3)	5.6 (3.8–9.3)	**0.002**	4.7 (3.6–7.5)	6.1 (3.8–9.1)	0.11

ALT, alanine transaminase; ALP, alkaline phosphatase; AST, aspartate aminotransferase; gGT, gamma-glutamyl transpeptidase; INSTIs, integrase strand transfer inhibitors; NK, natural killer cells; PIs/boosted, protease inhibitors/boosted. Significant *p*-values are in bold.

## Data Availability

The data that support the findings of this study are available from the corresponding author, M.P., upon reasonable request.

## Supplementary Materials

The following supporting information can be downloaded at: https://www.mdpi.com/article/10.3390/medicina60020331/s1, Table S1: commercially available kits used for the measurement of IL-6, sCD14, LBP, SuPAR, and I-FABP plasma levels.
