# Supplementary material for: Comparison of Integrase Strand Transfer Inhibitors (INSTIs) and Protease-Boosted Inhibitors (PIs) on the Reduction in Chronic Immune Activation in a Virally Suppressed, Mainly Male Population Living with HIV (PLWH)
Source: Medicina (Kaunas). 2024 Feb 15;60(2):331. doi: 10.3390/medicina60020331 (PMC10890512; doi:10.3390/medicina60020331)
Supplement: Supplementary file 1 [file medicina-60-00331-s001.zip › medicina-2816666-supplementary.pdf]

Comparison of integrase strand transfer inhibitors (INSTIs) and protease-boosted inhibitors (PIs) on the reduction in chronic immune activation in virally suppressed, mainly male population living with HIV (PLWH)

SUPPLEMENTARY FILE

Table S1. Commercially available kits used for the measurement of IL-6, sCD14, LBP, SuPAR, and I-FABP plasma levels.

The plasma levels of IL-6 were determined using a commercially available kit (Human IL-6; Mabtech, Inc, USA) with sensitivity 2 pg/ml, intra-assay 3,73%, and interassay coefficient of variation 6,3%.
The plasma levels of sCD14 were determined using a commercially available kit (Human sCD14; Wuhan Fine Biotech Co., Ltd., China) with sensitivity 0,094 ng/ml, intra-assay <8%, and interassay coefficient of variation <10%.
The plasma levels of LBP were determined using a commercially available kit (Human LBP; Wuhan Fine Biotech Co., Ltd., China) with sensitivity 18,75 ng/ml, intra-assay <8%, and interassay coefficient of variation <10%.
The plasma levels of SuPAR were determined using a commercially available kit (Human SuPAR; Wuhan Fine Biotech Co., Ltd., China) with sensitivity 0,094 ng/ml, intra-assay <8%, and interassay coefficient of variation <10%.
The plasma levels of IFABP/FABP2 were determined using a commercially available kit (Human IFABP/FABP2; Wuhan Fine Biotech Co., Ltd., China) with sensitivity 0,094 ng/ml, intra-assay <8%, and interassay coefficient of variation <10%.
